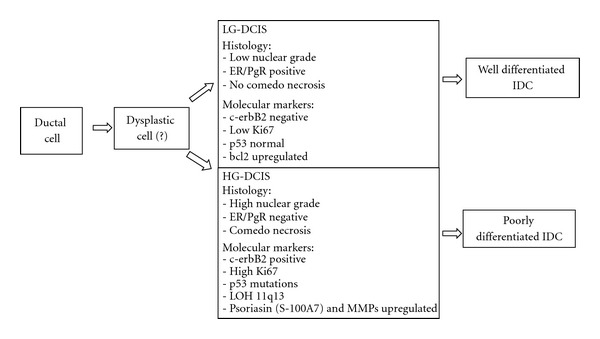# Intraductal Proliferative Lesions of the Breast—Terminology and Biology Matter: Premalignant Lesions or Preinvasive Cancer?

**DOI:** 10.1155/2012/501904

**Published:** 2012-05-10

**Authors:** Leopoldo Costarelli, Domenico Campagna, Maria Mauri, Lucio Fortunato

**Affiliations:** ^1^Department of Pathology, San Giovanni-Addolorata Hospital, Rome, Italy; ^2^Department of Medical Oncology, San Giovanni-Addolorata Hospital, Rome, Italy; ^3^Department of Surgery, San Giovanni-Addolorata Hospital, Rome, Italy

## Abstract

Morphological criteria for the diagnosis of intraductal proliferative lesions of the breast have been an object of research and much controversy, and its terminology is rather confusing. Knowledge of the molecular aspects of this disease probably necessitates further research to clarify if these entities can be identified as breast cancer precursors or as a malignant preinvasive disease. These issues are of great interest not only for their biological implications, but also to the clinician who must understand the disease and direct therapies. Molecular studies have shown that epitheliosis (usual ductal hyperplasia) is not monoclonal, while malignant lesions (atypical ductal hyperplasia, flat epithelial atypia, low-grade and high-grade intraductal carcinoma) constantly show these characteristics. These malignant lesions, classified with a DIN grading system (ductal intraepithelial neoplasia), are not obligate precursors of invasive ductal carcinoma and do not represent different evolving grades in a linear model of cancerogenesis. Breast cancerogenesis probably has different pathways with different morphologic precursors.

## 1. Introduction

Intraductal proliferative lesions (IPLs) of the breast are confined to the duct-lobular system, originating from the terminal duct-lobular unit (TDLU) with different cytological and architectural patterns of proliferation. They are characterized by an increase in the number of cells perpendicular to the basement membrane resulting in total alteration and distension of the normal unit structure of the breast without increasing in number [1].

Both a lobular and a ductal type of epithelial proliferation of TDLU are recognized. While the first type is quite monomorphic, intraductal proliferations show a wide variety of heterogeneous cytological aspects and architectural patterns. 

In the past several decades, there has been a wide discussion among pathologists worldwide regarding classification and grading of IPLs, with the aim to establish various risk categories. Despite these efforts, a high interobserver variability among pathologists regarding the diagnosis of IPLs as well established [2]. 

Criteria for diagnosis of IPLs by morphological means (Hematoxylin and Eosin and immunohistochemistry stains) are both qualitative (cytological and architectural changes), and quantitative. Diagnostic variability is not only due to interpretation of different patterns, but also to the difficulty in recognizing atypical cells isolated or in small clusters in TDLU. 

In this regard, there are several open questions in the literature, including the minimum threshold for grading, the risk of progression for different type of lesions, and the relationships between normal epithelium, IPLs, and invasive ductal carcinoma (IDC). Furthermore, it is unclear whether different types of IPLs are progressive steps of the same process or represent independent lesions leading to different malignancies. In the last few years, there have been numerous studies in this field, and several answers have been suggested to these questions.

These issues are of great interest not only to those involved in basic research, but also to the clinicians, because each type of lesion has different propensity for progression to local relapse and invasive disease. Therefore, understanding the biology of these lesions is paramount, and will contribute to a better delineation of appropriate guidelines for surgical treatment, as well as adjuvant and diagnostic recommendations.

## 2. Terminology and Historical Aspects

The term utilized by Azzopardi [3] for benign epithelial hyperplasia was *epitheliosis*. This term found little agreement among pathologists and has gained much wider acceptance in Europe than in North America. The alternative terms in the past were *papillary proliferation* and *papillomatosis*, because it can form “tongue-like” projection into ductal lumina, but without the connective core in papillae seen in papillary lesions of other organs. To date, epitheliosis appears a correct term because it includes the various patterns of benign proliferation, as fenestrated, solid, or papillary aspects. In recent years, the term *Usual Duct Hyperplasia* (UDH) [4] has been utilized for these lesions.

**Figure 1:**
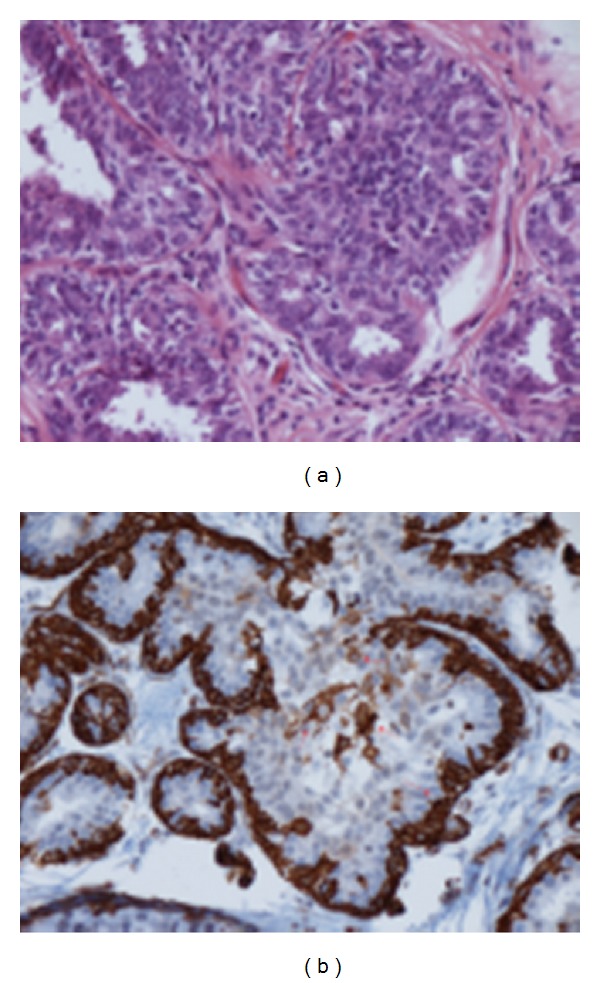
H.E. and immunoreaction for SM-actin—epitheliosis (usual ductal hyperplasia). Intraductal proliferation with irregular, “slit-like” lumina. The immunoreaction shows myoepithelial cells (*arrows*) surrounding the duct and in the proliferation.

Epitheliosis is a condition that has to be distinguished from in situ well-differentiated, low-grade carcinoma (LG-DCIS). The criteria for diagnosis have been well described by Azzopardi [3]. Two cell types are distinguishable in epitheliosis, epithelial, and myoepithelial, which have divergent differentiation. Immunohistochemical stains (p63, actin, calponin) are useful to detect myoepithelial cells in the lesion (Figures 1(a) and 1(b)). In epitheliosis, there is little distension of TDLU, few calcifications (not in granular form in necrotic debris), and absence of necrosis. A particularly complex pattern is the *infiltrating epitheliosis* [3, 5], previously described as *sclerosing adenosis with pseudo-infiltration* [6]. The so-called *sclerosing papillary proliferation* of Fenoglio and Lattes [7] probably represents the same entity. The hyperplastic epithelial structures are irregulars, distorted by an elastotic and sclerotic stroma. 

**Figure 2:**
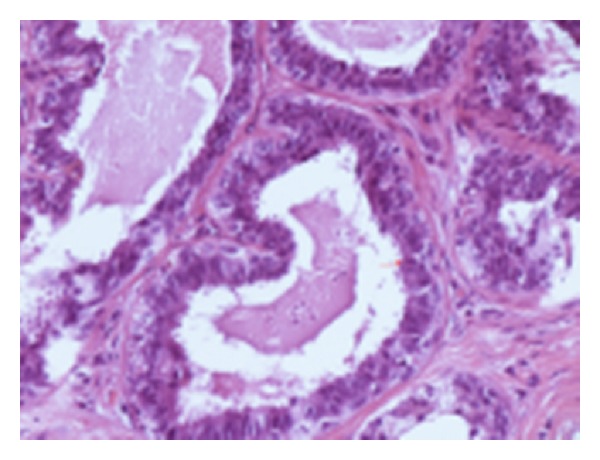
H.E.—flat epithelial atypia (DIN1a)—large TDLU with one to three layers of atypical ductal cells and mitosis.

**Figure 3:**
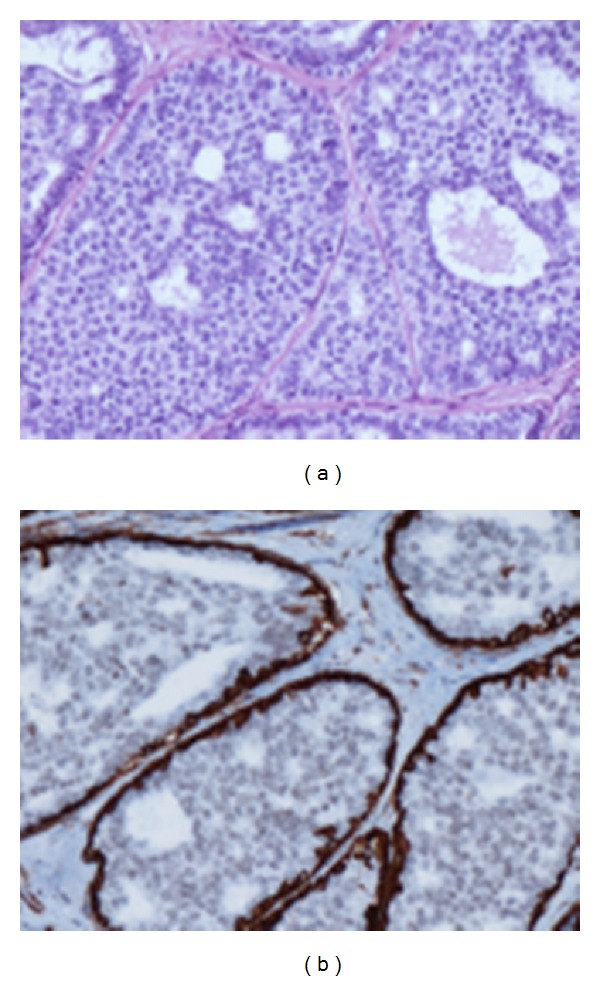
H.E and immunoreaction for SM-actin—low grade ductal in situ carcinoma (DIN1c). Cribriform type of intraductal proliferation with round, regular lumina, monomorphic round nuclei. Myoepithelial cells surround the duct but are not in the proliferation.

The hallmarks of LG-DCIS are architectural and cytological aspects. Myoepithelial cells cover the neoplastic ducts but are not present in the neoplastic proliferation (Figures 3(a) and 3(b)). Intraluminal or intraepithelial calcification, as calcific bodies, are frequent. The solid form of LG-DCIS has the same cytology. Another type of LG-DCIS was referred to by Azzopardi [3] as *type 2* or *monomorphous clinging carcinoma *(Figure 2). This pattern is particularly difficult to recognize, because the change is only cytological. One or few layers of columnar, atypical cells, define the lumen of variously larger TDLU. Pathologists have been much more reluctant to accept Azzopardi's second type of clinging carcinoma as a type of DCIS, particularly in the United States. This type of LG-DCIS is referred to also as *atypical cystic lobules*, *atypical lobules type A*, *atypical columnar change,* [8] and, in recent years, *flat epithelial atypia *(FEA) [4].

A “borderline” entity, between UDH and LG-DCIS, is controversial. Historically, there are two opposing approaches. The first viewpoint entails sharp demarcation between UDH and LG-DCIS, without intermediate lesions. Azzopardi said “…names like *atypical hyperplasia* should be avoided as far as possible.” The second viewpoint entails the existence of a continuum between hyperplasia and LG-DCIS, with different risk of progression for different grades of proliferation and atypia. Page et al. [9, 10] established two grades of hyperplasia, including *atypical hyperplasia*. Atypical hyperplasia is diagnosed when some features of LG-DCIS are present but other are lacking. When the duct is not completely involved, or when cytological appearance does not meet all the criteria of LG-DCIS. Other grading systems of IPLs suggested different grading of proliferation and atypia in borderline lesions [11]. The criteria proposed for the diagnosis of *atypical ductal hyperplasia* (ADH) are different: (i) lesions with cytological and architectural patterns of LG-DCIS are present, but both are not present in full flower; (ii) lesions with cytologic feature of LG-DCIS but lacking the typical growth pattern; (iii) lesions with cytological and architectural patterns of LG-DCIS but measuring in aggregate less than 2 mm [1]. Tavassoli [4] accepted the last criteria. 

**Figure 4:**
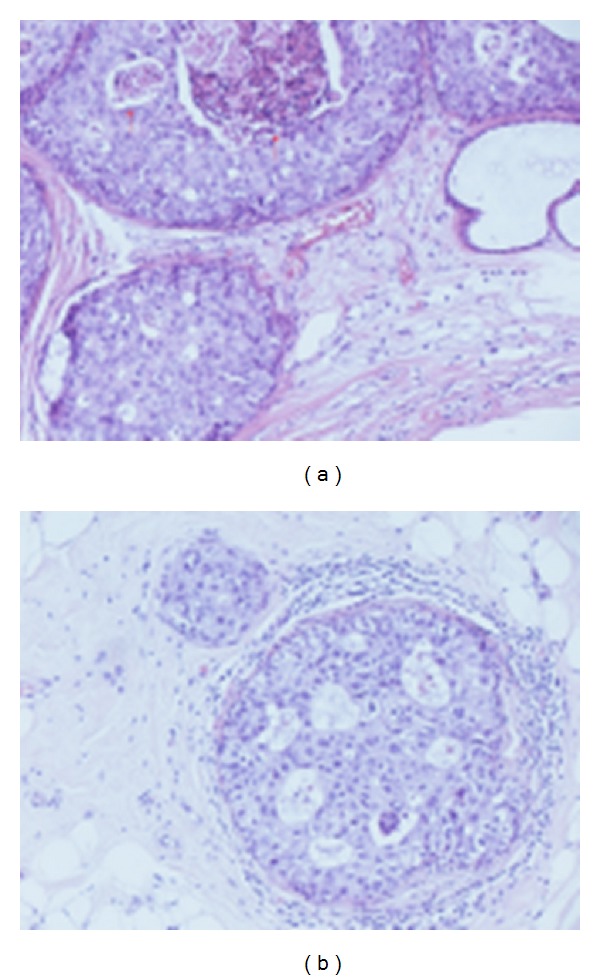
H.E.—intermediate grade ductal in situ carcinoma (DIN2). Obvious cribriform ductal in situ carcinoma with necrosis (Figure 4(a)—*arrow*) and intermediate cytological grade.

**Figure 5:**
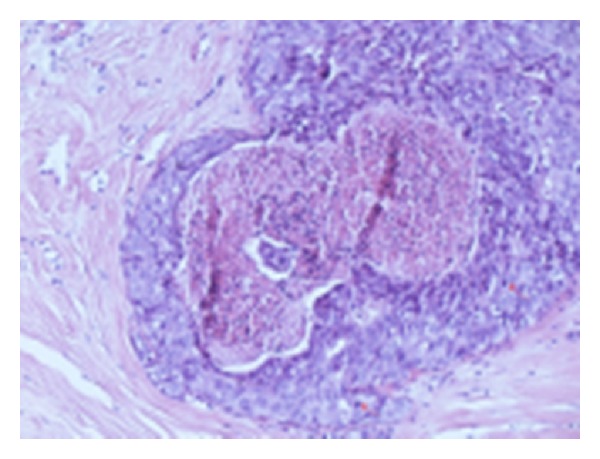
H.E.—high-grade ductal in situ carcinoma (DIN3). Solid intraductal carcinoma with high cytological grade and necrosis (*comedocarcinoma*) and numerous mitosis.

Intermediate grade DCIS (IG-DCIS) and high-grade DCIS (HG-DCIS) are obvious neoplastic lesions, graded by nuclear grade, necrosis, and architectural patterns. IG-DCIS has solid, papillary, or cribriform growth patterns, cytologically similar to those of LG-DCIS but with intraluminal necrosis (Figure 4(a)) or with intermediate cytological grade (Figure 4(b)), with or without necrosis. HG-DCIS has highly atypical cells proliferating as a single layer, or with papillary, cribriform, or solid pattern, usually with intraluminal necrosis. The HG-DCIS with solid pattern and large intraductal necrosis is referred to as *comedocarcinoma *(Figure 5). Typical granular calcifications on necrosis are present.

Rosai [2] proposed a terminology such as mammary intraepithelial neoplasia (MIN), like CIN of uterine cervix, for subjectivity and high degree of variability in interpretation of IPLs, disagreement about the criteria for definition of borderline lesions, and risk of progression of all types of IPLs. Afterwards this concept has been drawn as ductal intraepithelial neoplasia.

## 3. DIN System

Tavassoli [1] proposed to comprise all the IPLs in a single category, termed DIN, with various subtypes. This initial work by Tavassoli included epitheliosis or intraductal hyperplasia as DIN1a, because the risk of developing an invasive carcinoma was 1.5–2 times higher than in the general population. In the WHO book [4], UDH is separated by DIN, which includes FEA (DIN1A), ADH (DIN1B), LG-DCIS (DIN1C), IG-DCIS (DIN2), and HG-DCIS (DIN3) (Table 1). 

DIN System has some advantages. It diminishes the dualism cancer/no cancer, retains separation of all different subcategories but places LG-DCIS in the same group of ADH, because it considers the differences between these lesions quantitative, not qualitative. It eliminates the term cancer, diminishing the associated anxiety and emotional stress, and it incorporates the monomorphus clinging DCIS (FEA) in the same group of ADH and LG-DCIS.

The majority of the participants in the WHO Working Group were in favour of maintaining the traditional terminology with the new DIN System [4]. This fact shows well the disagreement and scepticism of pathologists.

## 4. Genetic and Molecular Findings

In the last twenty years, there have been numerous studies to search for genetic and molecular differences or similarities between the various forms of IPLs, heterogeneous in their cytological and architectural characteristics, and between IPLs and normal TDLU and IDC. Sometimes the results seem conflicting, but a more careful analysis reveals very interesting information about histogenesis, evolution, degree of progression, and invasiveness. The apparent contradictions are related to the different methods employed, with the difficulty of performing studies on very small lesions, with the possibility of contamination by normal tissue around the lesion. Laser capture microdissection has reduced these problems, and the possibility of using very advanced methods like comparative genomic hybridization on paraffin-embedded tissues has brought to utilize archive material [12].

A multitude of methods have been utilized: immunohistochemistry (IHC), in situ hibridization (ISH), analysis of loss of heterozygosity and allelic imbalance (AI), Comparative Genomic hybridization (CGH), cDNA microarrays (MA), and Proteomics Analysis (PA). The purposes of these methods are the study of growth characteristics, the expression of oncogenes, tumor suppressor genes, and other molecules, comparison of LOH and AI between the various forms of IPLs, and IPLs versus normal TDLU, IPLs versus IDC.

### 4.1. Growth Characteristics

The growth of any hyperplastic or neoplastic lesion is a balance between proliferation and cell death (apoptosis). Many researchers have studied the Proliferation Index (PI) with IHC, using antibodies marking cellular proliferative cycle like Ki67 [13]. In premenopausal women, the PI of the normal TDLU varies in different phases of the menstrual cycle. In luteal phase, it is higher than in proliferative phase. The median PI in normal TDLU is 2%, in ADH it is 5%, in DCIS it is 5%. LG-DCIS and FEA have a median PI of 5%, while in HG-DCIS it is 20%. In any case there is a continuous range from 1% and 70% from very well and poorly differentiated lesions.

ADH has a lower rate of apoptosis (0,3% versus 0,6%) and higher PI than normal TDLU; DCIS has higher apoptosis (5%) and PI than normal TDLU and ADH. Apoptosis varies from 1% to 5% among LG-DCIS and HG-DCIS, and it represents a continuous variable [14]. Higher cellular death in high-grade lesions means that the growth is a complex phenomenon. HG-DCIS has large positive growth imbalance with a high cellular death. Disturbance of the equilibrium between cell proliferation and cell death is the result of numerous alterations of growth regulating mechanisms involving sex hormones, oncogenes, and tumor suppressor genes.

### 4.2. Sex Hormones, Oncogenes, and Tumor Suppressor Genes

Estrogens, through Estrogen Receptor (ER), play a central role in growth and differentiation of normal TDLU epithelium, stimulating cell proliferation and regulating expression of other genes, like Progesteron Receptor (PgR) [15]. Two types of ER are described: ER*α* and ER*β*. ER*α* is the more studied molecule. 90% of normal TDLU express ER in an average 30% of cells [14]. There is a change of expression during the menstrual cycle: in the luteal phase, the rate of positive cells is higher (40% versus 20%). In postmenopausal women, the rate is higher (50%). Nearly all cells of ADH express ER. ER is expressed in 75% of DCIS; 100% in LG-DCIS, nearly all cells, and 30% in HG-DCIS, usually in a rate of cells [14, 16]. 

Many molecules are studied in IPLs, but the majority of studies have not been validated [17]. Exceptions are c-erbB2 (*neu*) and p53.

In IDC, c-erbB2 (*neu*) is overexpressed or amplified in 10–20% of cases, generally HG-IDC. It plays a role in cell proliferation, is related to poor clinical outcome, is a predictive marker for responsiveness to various therapies, and promotes cell mobility [18]. In recent years, it has been one of the more studied markers in breast cancer, because it is a target for trastuzumab therapy. Normal TDLU, UDH, ADH, and FEA do not express c-erbB2 (*neu*). LG-DCIS and intermediate grade express neu in less than 10% of cases, whereas in HG-DCIS it is overexpressed in 60% of cases [19]. 

The more utilized method to detect expression of p53 is IHC, which is a surrogate assay for detecting mutations, because a gene with missense mutations codifies for inactivate protein. This abnormal inactive protein is accumulated in very high levels in the nucleus of neoplastic cells, and it is detectable by IHC [20]. 30% of IDC overexpress p53, and it is related to aggressive biological features and poor clinical outcome. Normal TDLU, UDH, and ADH do not overexpress p53, apart from in the Li-Fraumeni syndrome, characterized by inherited mutations. In DCIS p53 correlates with differentiation. It is rare in LG-DCIS and common (40%) in HG-DCIS [21].

Clark et al. [22] found by IHC analysis on tissue microarray (TMA) the same molecular classes in DCIS as in IDC [23, 24], but with different frequency. Basal-like phenotype is rare in DCIS; c-erbB2 is more frequently expressed in Luminal-like DCIS than in IDC (13,2% versus 5,2%). The mitochondrial antiapoptotic protein *bcl2* is up-regulated in LG (G1-G2) Luminal-type DCIS. The same results are in the study of Tamimi et al. [25]. Livasy et al. [26] found a higher frequency of Basal phenotype in DCIS.

Other genes and proteins are studied in IPLs and compared with IDC. 

Ma et al. [27] studied five genes with quantitative RT-PCR in ADH and DCIS, upregulated or downregulated in IDC. These genes were altered in the same rate in ADH, DCIS, and IDC: for example, CRIP1 was upregulated in 7/8 ADH, in 27/30 DCIS and in 23/25 IDC, and ELF5 was downregulated in 7/8 ADH, in 28/30 DCIS and 25/25 IDC. Significant alterations in gene expression of ADH are maintained in the later stages of DCIS and IDC. Furthermore, to characterize the molecular link between DCIS and IDC, the study recognizes other clusters of genes related to infiltrative potential. These genes establish different patterns of gene expression among DCIS, which reflect different invasive potential, and there is a gene signature of DCIS, like for IDC [28].

Gillett et al. [29] studied expression of Cyclin D1 in ADH and DCIS. In IDC, expression of Cyclin D1 is more frequent in well differentiated and ER+ cases. ADH does not express Cyclin D1, but it is ER+ like LG-DCIS: in DCIS Cyclin D1 is over-expressed in 64% of cases, and it is not related to grade or to ER expression.

Schuetz et al. [30] studied genes of epithelial mesenchymal Transition (EMT) and other genes candidates to cause invasion and metastases [31]. These genes (Twist1, SPARC, MMP13, MMP11, BPAG1) are markers of transition from DCIS to IDC. Some of the proteins codified by these genes are very interesting: for example, BPAG1 is expressed in hemidesmosomes connecting epithelial cells to basement membrane. IDC cells do not contain hemidesmosomes and BPAG1 is downregulated. Matrix metalloproteinases (MMPs) can degrade different components of extracellular matrix, including laminin, fibronectin, collagen, and elastin, and are upregulated in HG-DCIS.

Porter et al. [32] and Kretschmer et al. [33] found several genes of IDC up-regulated in DCIS using SAGE (Serial Analysis of Gene Expression), TMA, quantitative RT-PCR, and IHC. Some of these genes are not related to differentiation grade (MUC1, SBP1) and probably play a role in early cancerogenesis. Other proteins, like the psoriasin (S-100A7), a calcium-binding protein that regulates cell cytoskeleton and motility, are present in comedo-type HG-DCIS.

### 4.3. LOH and Allelic Imbalance (AI)

Two types of studies are performed: the first on IPLs in their pure form and the second on IPLs with synchronous IDC in the same breast. The strategy of the last type is to identify alterations, which may be important in the invasiveness [17]. Generally, IPLs with synchronous invasive cancer share more frequent genetic alterations with IDC than pure forms. For example, a marker on chromosome 11p (D11S988) is more frequent in all IPLs close to IDC than in IPLs without cancer [34]: morphologically normal TDLU close to IDC also shared rarely some LOH with cancer [35]. O'Connell et al. [34], assessing LOH to 15 loci on 12 chromosomes, found that 50% of ADH shared their LOH phenotypes with synchronous IDC, providing novel and compelling genetic evidence that ADH is a direct precursor of IDC. Many studies of DCIS have shown that nearly all lesions share several identical AI with synchronous IDC, providing convincing if not surprising evidence that they are evolutionarily related too [34–38]. Synchronous DCIS and IDC may occasionally show distinct AIs, suggesting that there may also be divergent aspects to their evolution [25].

There are numerous studies on LOH in IPLs without invasive cancer. 

Allelic Imbalance in UDH is rare: Lakhani et al. [39] found alterations in 0–13% of studied loci, frequently in 17q; Deng et al. [35] found alterations in different loci in 0–15%. 

Moinfar et al. [40] found AI on 77% of FEA, at least in one locus 11q, 16q, and 3p. 11q and 16q are frequently involved in tubular carcinoma.

Morphological overlap between ADH and LG-DCIS is reflected at molecular level. Up to 50% of ADH contains one or more AIs among 30 genetic loci [35, 39], and they are the same of DCIS. 

CGH analysis of DCIS has demonstrated a large number of alterations, including gains of 1q, 5p, 6q, 8q, 17q, 19q, 20p, 20q, and Xq, and losses of 2q, 5q, 6q, 8p, 9p, 11q, 13q, 14q, 16q, 17p, 17q, and 22q [35, 41, 42]. Some AI are more frequent and constitute hot spots: loss in 11q, 16q, 17p and 17q.

Rosenberg et al. [43] studied a series of 15 microsatellite loci in ADH and found monoclonal microsatellite alterations in 40% of cases in more than one locus, suggesting that a genetic instability plays an early role in cancer progression.

Wiechmann and Kuerer [44] characterize the differences between LG-DCIS and HG-DCIS and their risk of progression in IDC by means of the expression of steroid receptor (LG-DCIS is frequently ER/PgR positive), growth characteristics (Ki67 is lower in LG-DCIS than in HG-DCIS), expression of c-erbB2 (frequent in HG-DCIS, rare in LG-DCIS), bcl2 and p53 (the first over-expressed in LG-DCIS, the second frequently mutated in HG-DCIS), expression of psoriasin (S-100A7) and metalloproteinases (MMPs) (upregulated in HG-DCIS), and allelic imbalance (LG-DCIS has frequently gain of 1q and loss of 16q, whereas HG-DCIS shows frequently 17q12 and 11q13 amplification).

In Table 2 are reported the more frequent alterations in IPLs and IDC.

## 5. Discussion

One of the most controversial topics about breast pathology concerns IPL, with a wide range of phenotypic manifestations from epitheliosis to DCIS. Page et al. [9, 10] have suggested that there is a continuum between these two extremes, with an intermediate condition called ADH. Azzopardi [3], on the other hand, draws a sharp line of demarcation between hyperplastic and neoplastic lesions, and he stigmatizes: “…names like “atypical hyperplasia” should be avoided as far as possible”. He himself describes a particular type of DCIS called clinging carcinoma. The low-grade clinging carcinoma (type 2 according to Azzopardi) and columnar change is named also flat epithelial atypia (FEA). 

High interobserver variability among experienced pathologists in ADH interpretation is reported [2], mainly related to different proposed criteria. WHO book [4] suggests the use of dimensional criteria of Tavassoli [1]: ADH is a lesion with cytological and architectural pattern of LG-DCIS measuring in aggregate less than 2 mm.

The various IPLs have different risk of progression: UDC has two folds, ADH four folds, and LG-DCIS ten folds compared to normal breast [9, 10]. At the molecular level, rarely UDH shows allelic imbalance (AI) for one gene, whereas ADH, FEA, and LG-DCIS show frequent AIs for many genes [34–39]. Expression of high-molecular-weight cytokeratin is different between UDH and other IPLs: UDH consistently displayed the presence of a population of cytokeratin 5/6-expressing basal-type cells within the proliferative lesion, whereas ADH and LG-DCIS lacked cytokeratin 5/6-positive cells. A subset of HG-DCIS express cytokeratin 5/6: it is probably the precursor of Basal-like IDC [42]. The studies about growth characteristics (proliferative index and apoptosis) and about expression of different molecules like ER, oncogenes, and tumor suppressor genes display a substantial difference between UDH and other IPLs. ADH, FEA, and LG-DCIS, on the other hand, shares many biological characteristics with IDC [13, 14, 16, 17, 45], and microsatellite analysis shows monoclonality and genetic instability in ADH [43]. This supports the concept that UDH is a not malignant lesion, the opposite to other IPLs. ADH, FEA and LG-DCIS can be set in the same group of pre-invasive breast neoplastic lesions. DIN system, as well as proposed by Tavassoli [4], includes ADH, FEA and DCIS, not UDH. 

Several data support the concept that different types of DCIS show different genetic alterations [41, 42]. Alterations at 16q are much more frequent in LG-DCIS than in HG-DCIS, in which alterations at 13q, 17q, and 20q are more frequent [4, 6, 7, 10]. Similar findings are in invasive carcinomas of low and high grade [41, 42, 46–48]. On the other hand, LG-DCIS share many molecular alterations with ADH [35, 39] and LG-IDC, and also the few studies on FEA [40] show alterations similar to LG-IDC, in particular with a very well-differentiated IDC (tubular carcinoma). These molecular studies reflect the same morphological findings: (i) it is extremely rare to find an HG-DCIS in a LG-IDC, as well as a LG-DCIS in an HG-IDC; (ii) in tubular carcinoma we see frequently an in situ component like FEA or LG-DCIS cribriform type. Also, growth characteristics and rates of expression of sex hormones, oncogenes, and tumor suppressor genes suggest that LG-DCIS is a precursor of LG-IDC and HG-DCIS is a precursor of HG-IDC [41, 42]. These data suggest that there may be multiple pathways for the evolution of IPLs and IDC. In various tissues, a linear multistep progression between various preinvasive stages, which end in invasive cancer, is recognizable: in colonic mucosa, for example, there is a linear multistep model from normal epithelium to invasive carcinoma through the sequence hyperproliferative epithelium, adenoma, carcinoma, and any morphological step is related to a specific genetic alteration [49]. In breast cancer, the linear model undoubtedly oversimplifies a complex process. There is no morphological or molecular evidence that LG-DCIS progress into an HG-DCIS, or into HG-IDC. The model that results from morphological and molecular data is horizontal (or parallel), and it is done by two or several pathways (Table 3).

For this reason, the DIN system is not a progression through different grades, like the intraepithelial neoplasia in other tissue (e.g., the CIN system of cervical cancer), but a classification of different intraductal neoplastic conditions, each of these are not an obligate precursor of IDC.

The DCIS classification of Wiechmann and Kuerer [44], based on biological potential to progress into IDC, shows two parallel pathways of progression with different histologic characteristics and molecular markers. Between these two pathways, there is a presumptive common progenitor, a *dysplastic cell*, not better characterized (Table 4).

In IDC, recent studies have led to a molecular classification based on the biological characteristics of the tumor rather than limited to morphological analysis. Perou [23] and Sotiriou et al. [24] identified molecular subtypes of invasive breast cancer based on an intrinsic gene signature. Many studies have aimed to identify an IHC profile that can act as a surrogate for gene array analysis, and it appears that a five-marker panel of estrogen receptor (ER), progesterone receptor (PR), c-erbB2, cytokeratin 5/6, and EGFR shows ability to categorise invasive cancers to their molecular subtype [50]. Much less attention has been focused on dissecting the biological subtypes of DCIS, and there are discrepancies in the results. Thus, whereas several studies report the existence of a basal subtype of DCIS [25, 51], one gene array study found no firm evidence of this category of DCIS or it is much less frequent [51]. There are also other discrepancies in the relative frequency of subtypes between the in situ and invasive disease. It has been recognized, for example, that there is a higher frequency of c-erbB2-positive DCIS compared with c-erbB2-positive IDC [52]. The difference in expression of c-erbB2 is actually inexplicable: the hypotheses advanced are that the expression is switched off during invasion or that many c-erbB2-positive DCIS do not transform to IDC.

## 6. Conclusions

Diagnosis and reproducibility of proposed criteria of IPLs are complex. This is testified by terminological confusion, with a large number of designations for the same entity. To render the issue even more controversial, it is not clear whether some of these entities really occur in practice. Interobserver agreement in diagnosis of intermediate lesions is low. The DIN system unifies the terminology, while it may have the additional advantage to decrease the anxiety and emotional stress of patients.

Morphological complexity is reflected by the large variety of molecular findings described by a number of studies in the last twenty years. Some of these alterations are confirmed by different studies, and their comparison with different clinical entities provides much information about their nature and propensity for progression. UDH or epitheliosis is probably a benign process, while other IPLs (ADH, LG-DCIS, HG-DCIS, FEA) are neoplastic processes. ADH and FEA shares many alterations with LG-DCIS and LG-IDC, while HG-DCIS share many alterations with HG-IDC. Probably, the model of breast carcinogenesis is more complex than in other tissues, because the results of the molecular studies suggest parallel different pathways of carcinogenesis. By this point of view, the DIN system is not a progression through different additional steps, like the intraepithelial neoplasia in other tissues (for example, the CIN system for uterine cervix cancer), but a classification of different conditions, which are not obligate precursors of IDC.

## Figures and Tables

**Table 1:** Terminology of IPLs.

DIN system	Traditional terminology
Usual ductal hyperplasia (UDH)	Epitheliosis Infiltrating epitheliosis Papillomatosis Sclerosing adenosis with pseudoinfiltration Sclerosing papillary proliferation

DIN1a	Clinging carcinoma monomorphus or type 2 Flat epithelial atypia Atypical cystic lobules Atypical lobules type A Atypical columnar change

DIN1b	Atypical ductal hyperplasia (ADH)

DIN1c	Low-grade ductal carcinoma in situ (LG-DCIS) DCIS grade 1

DIN2	Intermediate-grade ductal carcinoma in situ (IG-DCIS) DCIS grade 2

DIN3	High-grade ductal carcinoma in situ (HG-DCIS) DCIS grade 3

**Table 2:** “hot spots” in IPLs—more frequent allelic imbalances reported.

	Gains	Losses
UDH	rare	rare
ADH	1q, 16p	11p, 11q, 16q, 17p
FEA	—	3p, 11q, 16q
DCIS	1q (*LG-DCIS*), 5p, 6q, 8q, 17q, 19q, 20p, 20q *(HG-DCIS)*, Xq	2q, 5q, 6q, 8p, 9q, 11q (*HG-DCIS*),13q *(HG-DCIS)*, 14q, 16q *(LG-DCIS)*, 17p, 17q *(HG-DCIS)*, 22q

**Table 3:** Cancerogenesis models.

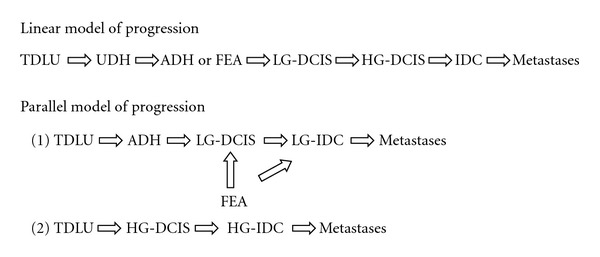

**Table 4:** Classification of DCIS based on biologic potential [44].